# In Situ Real-Time Monitoring of Glutamate and Electrophysiology from Cortex to Hippocampus in Mice Based on a Microelectrode Array

**DOI:** 10.3390/s17010061

**Published:** 2016-12-30

**Authors:** Xinyi Fan, Yilin Song, Yuanlin Ma, Song Zhang, Guihua Xiao, Lili Yang, Huiren Xu, Dai Zhang, Xinxia Cai

**Affiliations:** 1 State Key Laboratory of Transducer Technology, Institute of Electronics, Chinese Academy of Sciences, Beijing 100190, China; xinyi_fan@126.com (X.F.); ylsong@mail.ie.ac.cn (Y.S.); zhangsong0112@126.com (S.Z.); xiaoguihua11@126.com (G.X.); lilisheepmiemie@163.com (L.Y.); xuhuiren_2012@163.com (H.X.); 2 University of Chinese Academy of Sciences, Beijing 100190, China; 3 Institute of Mental Health, the Sixth Hospital of Peking University, Beijing 100191, China; mayuanlin2010@bjmu.edu.cn (Y.M.); daizhang@bjmu.edu.cn (D.Z.)

**Keywords:** MEMS, implantable microelectrode array, glutamate, spike, hippocampus

## Abstract

Changes in the structure and function of the hippocampus contribute to epilepsy, schizophrenia and other neurological or mental disorders of the brain. Since the function of the hippocampus depends heavily on the glutamate (Glu) signaling pathways, in situ real-time detection of Glu neurotransmitter release and electrophysiological signals in hippocampus is of great significance. To achieve the dual-mode detection in mouse hippocampus in vivo, a 16-channel implantable microelectrode array (MEA) was fabricated by micro-electromechanical system (MEMS) technology. Twelve microelectrode sites were modified with platinum black for electrophysiological recording and four sites were modified with glutamate oxidase (GluOx) and 1,3-phenylenediamine (mPD) for selective electrochemical detection of Glu. The MEA was implanted from cortex to hippocampus in mouse brain for in situ real-time monitoring of Glu and electrophysiological signals. It was found that the Glu concentration in hippocampus was roughly 50 μM higher than that in the cortex, and the firing rate of concurrently recorded spikes declined from 6.32 ± 4.35 spikes/s in cortex to 0.09 ± 0.06 spikes/s in hippocampus. The present results demonstrated that the dual-mode MEA probe was capable in neurological detections in vivo with high spatial resolution and dynamical response, which lays the foundation for further pathology studies in the hippocampus of mouse models with nervous or mental disorders.

## 1. Introduction

Glutamate (Glu) is an important excitatory neurotransmitter in the mammalian central nervous system (CNS), and it is involved in various aspects of brain function [1]. For example, the hippocampus is part of the limbic system, and its normal function is heavily dependent on glutamate-signaling pathways. Since neurons in the CNS communicate both electrically and chemically [2], in situ real-time monitoring of Glu and electrophysiological signals in the hippocampus is of great significance to comprehensively understand neuronal states and the mechanism of the Glu signaling system, which will further contribute to the effects underlying the pathogenesis of related nervous or mental diseases [3,4].

Some previous works have implemented hippocampus dual-mode studies in vivo by combining separate approaches: implanting electrodes to record electrophysiological signals while adopting high-performance liquid chromatography (HPLC) to detect Glu release [5,6]. However, the minute-level time resolution of HPLC cannot meet the second-by-second real-time detection demands [7]. Also, some reports have focused on electrophysiological studies in the hippocampus by using patch-clamp techniques or silicon microelectrode arrays, however, they did not realize simultaneous Glu detection [8,9].

Recent advances in micro-electromechanical system (MEMS) technology have promoted the development of microelectrode arrays (MEAs) with high resolution and high sensitivity for neuronal research [2,9,10,11], and microelectrodes for measurements of L-glutamate and other neurotransmitters have been reported with excellent rejection of cross-talk and interference by using self-reference techniques [12,13]. In this study, we prepared a 16-channel implantable MEA integrating 12-channel electrophysiological recording sites and 4-channel electrochemical recording sites. The MEA probe was acutely implanted from the cortex to the hippocampus in anesthetized mice. In situ distribution of inherent Glu concentrations and spontaneous electrophysiological signals, including local field potentials (LFPs) and action potentials (spikes), were simultaneously recorded in layered brain regions. The result demonstrated the feasibility of the probe for dual-mode detection in vivo and laid the foundation of further pathology studies in mouse models of nervous or mental disorders.

## 2. Materials and Methods

### 2.1. MEA Probe Fabrication and Preparation

**Figure 1:**
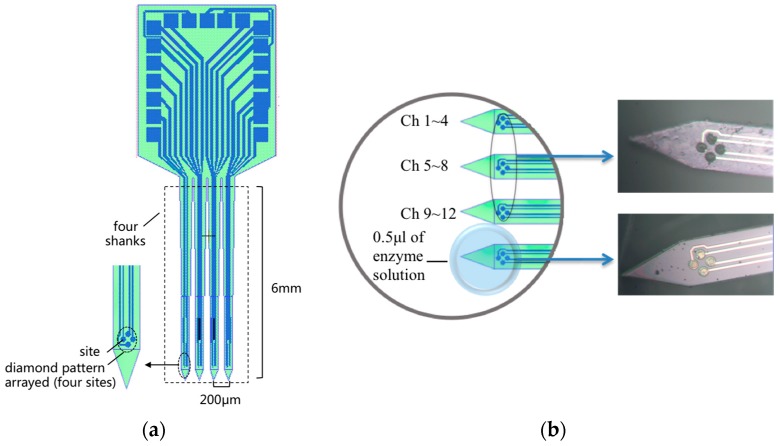
(**a**) Sketch map of the 16-channel (4 × 4) implantable microelectrode array; (**b**) Schematic illustrates the enzyme coating process with micromanipulator under microscope. Channels (Ch) 1–12 were deposited with platinum black for electrophysiology detection, and Channels 13–16 were modified with D-glutamic oxidase-m-dihydroxybenzene (GluOx-mPD) for glutamate flux detection.

Silicon-based MEA was developed on silicon-on-insulator (SOI, thickness: 30 μm Si/1.5 μm SiO_2_/600 μm Si) wafer based on MEMS techniques as described previously [14,15,16,17]. The MEA probe constructed for our mouse study consisted of four 6 mm long, 100 μm wide, 30 μm thick shanks with spacing of 200 μm between the shanks. On tips of the shanks, 16-channel thin film platinum sites (10 μm in diameter) were 4 × 4 arrayed with a diamond pattern on each tip (as shown in Figure 1a).

Channels 1–12 on three adjacent shanks were electrodeposited with nano-Pt to form electrophysiological recording sites with low impedance. To achieve directional enzyme coating on Glu recording sites (Channels 13–16) on the left marginal shank, the MEA was fixed on a tri-axial micromanipulator and the coating process was completed under a microscope: 0.5 μL solution of glutamate oxidase (GluOx) containing a final concentration of 1% bovine serum albumin (BSA) (≥99%, Amresco, Solon, OH, USA), 0.125% glutaraldehyde (25%, Shanghai Chemical Reagent Company, Shanghai, China), and 1% GluOx (Yamasa Corp., Tokyo, Japan) was drawn up and formed a drop of solution on parafilm under a dissecting stereoscope (as shown in Figure 1b), then the tip of the marginal shank was slowly manipulated into the bead for dip-coating three times. The BSA and glutaraldehyde helped induce cross-linking of the GluOx onto the platinum recording sites. Next, a solution of 5 mM·mPD (≥99%, Sigma-Aldrich, Taufkirchen, Germany) was dissolved in phosphate-buffered saline (PBS, 0.01 M, Na_2_HPO_4_-NaH_2_PO_4_-KCl, pH 7.4, Sigma-Aldrich) and then electrochemically deposited on Channels 13–16 to prevent interference from endogenous electroactive molecules such as ascorbic acid, dopamine and serotonin. The MEA was cured at room temperature for 48–72 h prior to calibration.

**Figure 2:**
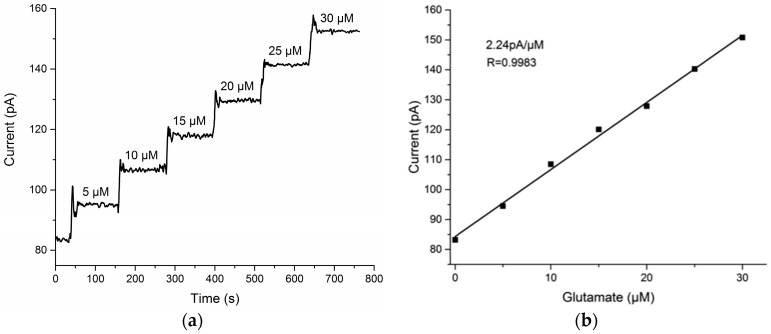
(**a**) I-T curve, voltage 0.7 V, response of the GluOx-mPD modified recording site on the MEA to Glu solution with concentrations of 5–30 μM; (**b**) Linear fit of the Glu oxidation currents, the sensitivity is 2.24 pA/μM, *R* = 0.9983.

The Glu recording sites were calibrated at a constant potential of +0.7 V versus Ag|AgCl electrode to determine their sensitivity to Glu concentration, at which potential the reporter molecule H_2_O_2_ would be oxidized. The constant potential amperometry was performed using a potentiostat (Reference 600, Gamry Instruments, Warminster, PA, USA). The tip of the MEA was placed in a continuously stirred solution of 0.05 M PBS at 37 °C. Glu solution with high concentration was s added step-wise into the buffer to make aliquots of 5, 10, 15, 20, 25 and 30 μM for calibrations. As illustrated in Figure 2, the current increased as the Glu concentration rose, and the sensitivity was calculated from the slope of the linearity. Reported as mean ± S.E.M., the four Glu recording sites had average slopes of 2.24 ± 0.03 pA/μM with relative coefficient of 0.9983.

### 2.2. In Vivo Experiments

A 21-week-old male mouse weighing 25–30 g (Institute of Mental Health, Peking University Sixth Hospital, Beijing, China) was anesthetized with pentobarbital sodium (0.7%, Fluka, Taufkirchen, Germany) and placed in a stereotaxic frame. The mouse underwent scalp incision and a 1 mm × 1 mm craniotomy over the left hippocampus (AP: −2–−3 mm, ML: 1.5–2.5 mm) for MEA implantation. Another craniotomy was conducted on the left hemisphere symmetrically for an Ag|AgCl wire implantation as reference electrode. A bone screw was fixed into the skull as ground and the mouse with the stereotaxic frame were placed inside a grounded Faraday cage.

**Figure 3:**
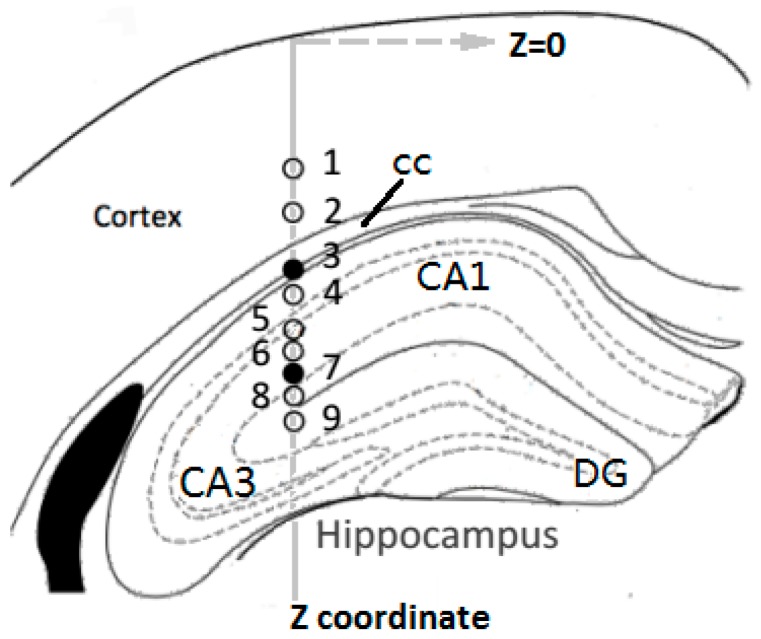
The MEA implantation path from cortex to hippocampus in mouse brain (coronal plane view): The gray dotted lines outlined different parts of the compact pyramidal neuron layer in hippocampus. *Z* coordinates of the nine targeted depths numbered from 1 to 9 were, in sequence, 0.8, 1.0, 1.2, 1.3, 1.5, 1.6, 1.7, 1.8 and 1.9 mm.

Prior to implantation, the dura mater was resected and the MEA tip was lowered down by a manual hydraulic probe drive (50-12-1C, FHC, Bowdoin, ME, USA) to contact the pia mater, and this position was marked as zero point on *Z* coordinate (Figure 3). The drive could control the vertical movements of the MEA probe with a precision of 2 μm. A drop of PBS was added onto the pia mater around the probe tip, and baselines of both electrophysiological and electrochemical signals were recorded for 30 min. Then, with the concurrent dual mode recordings continued, the MEA probe was slowly inserted into brain tissue at a nearly constant rate of about 5 μm/s. The longitudinal distance of implantation path was 2 mm, across the cortex to hippocampus. In order to compare the features of neural activities from different layered structures in mouse brain, nine targeted depths (*Z* = 0.8 mm, 1.0, 1.2, 1.3, 1.5, 1.6, 1.7, 1.8 and 1.9 mm) were particularly concerned, at which the MEA would be stopped and stayed for about 30 s to acquire in situ signals. All procedures above complied with the guidelines of State Scientific and Technological Commission for the care and use of laboratory animals.

For dual-mode real-time recording, the microelectrode 1~12 on the MEA probe were separately connected to 12 channels of the electrophysiological recordings system (USB-ME16-FAI-System, MultiChannel Systems, Reutlingen, Germany). The 12-channel signals were sampled at the rate of 30 kHz, with low pass filter of 250 Hz to view LFPs and band pass filter of 500 Hz–5 kHz to view spikes [14]. Meanwhile, one of the microelectrodes 13–16 was connected to the single channel potentiostat (Reference 600, Gamry Instruments, Warminster, PA, USA). Using chronoamperometry method, a constant working potential of +0.7 V (versus the Ag|AgCl wire reference electrode) was applied, and the electrochemical current was sampled at the rate of 2 Hz.

## 3. Results and Discussion

### 3.1. Glutamate Recordings from Cortex to Hippocampus

**Figure 4:**
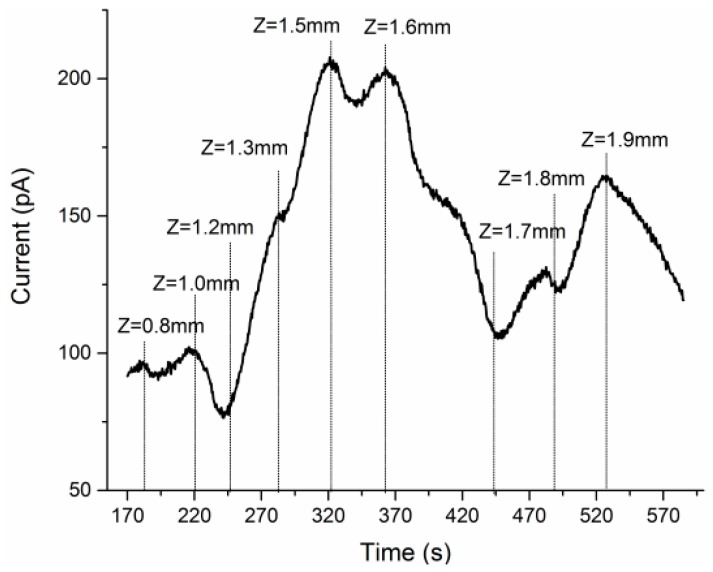
The glutamate oxidation current observed from the mPD-GluOx modified sites in the process of MEA implantation from cortex to hippocampus.

In the beginning of implantation, the baseline current recorded by the Glu recording site finally stabilized at about 15.19 pA at *Z* = 0 mm. Figure 4 showed the amperometric I-T curve recorded from the cortex to the hippocampus (*Z* = 1.9 mm) during implanting the MEA over a period of about 400 s. The nine targeted depths were read out from the hydraulic probe drive and marked with vertical dotted lines in the figure.

As shown in Figure 4, compared to the baseline current (15.19 pA), the Glu oxidation current increased to about 90–100 pA in cortex (*Z* = 0.8 mm–1.0 mm). When divide this increment (about 80 pA) by the previously calibrated sensitivity (2.24 pA/μM), the estimating concentration of Glu reached 35 μM in cortex. The Glu here was mainly secreted by the large pyramidal neurons in Layer V of the cortex. After that, tip of the MEA moved down to the brain structure of corpus callosum (cc) (*Z* = 1.0 mm–1.2 mm), which consisted of neural fiber bundles and filled up the spaces between the cortex and hippocampus.

There are no neurons in the cc, so the Glu oxidation current gradually decreased. As the implantation continued, there was a significant rise in glutamate concentration after the MEA penetrated the border of hippocampus, and the I-T curve displayed several peaks and valleys reflecting the inhomogeneous distribution of Glu in layered structures of hippocampus. It was reported that, the hippocampus region is mainly made up of a compact layer consisting of 5–8 superimposed rows of pyramidal neurons [18], and these neurons mainly release glutamate as excitatory neurotransmitter [19]. As shown in Figure 3, he pyramidal neuron layer folded and formed different parts called CA1, CA3, DG, etc., and neural fibers filled most of the spaces between the folds. Such sandwich construction led to the inhomogeneous distribution of Glu, and the MEA could respond to the changing of Glu concentration with high special resolution in real time. The specific concentration values would be calculated in Section 3.3.

### 3.2. Electrophysiological Recordings from Cortex to Hippocampus

**Figure 5:**
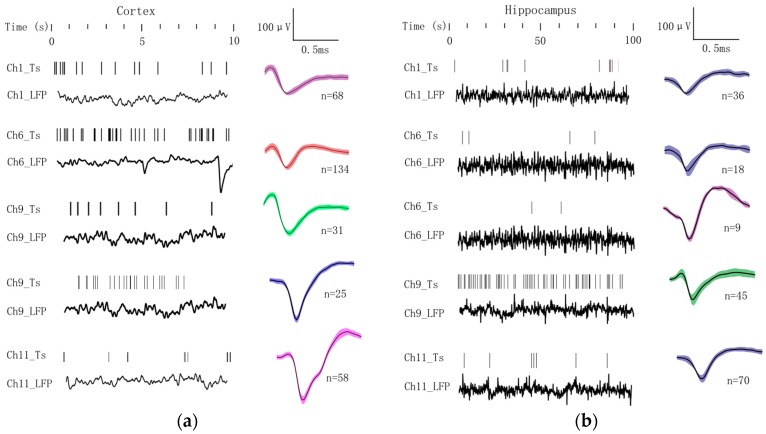
Spike trains and LFPs signals sampled from (**a**) cortex; and (**b**) hippocampus with four electrophysiology sites on MEA, the corresponding waveforms and spike counts are listed on the right column (each typical spike type is indicated with one color).

Although the mouse was anesthetized to be unconsciousness, and neural activities were partially inhibited, there were still millions of neurons that remain active to maintain basic neurophysiological functions. These neurons would spontaneously fire action potentials (spikes) and release different neurotransmitters to communicate with each other, and the summed action potentials from multiple nearby neurons within a small volume of brain tissue generated local field potential (LFP). To investigate such neural activities from anesthetized mouse, electrophysiological recordings were made during the implantation from cortex and hippocampus. Typical electrophysiological signals recorded by four sites (Channels 1, 6, 9 & 11) on the four separate shanks were shown in Figure 5. Action potentials from single or multi neurons were discriminated using K-means clustering analysis by Offline Sorter software (Plexon Inc., Dallas, TX, USA). Then spike timestamps and unfiltered LFPs data were imported into NeuroExplorer software (Nex Technologies, Madison, AL, USA) for further analysis.

There were distinct differences on features of electrophysiological signals between cortex and hippocampus. In Figure 5a, the four-channel spikes and LFPs in cortex were displayed over a period of 10 s, and spike waveforms and counts were shown on the right column respectively. Five typical spike types were recorded with Channel 9 detecting two distinct types. The spike firing rates in cortical areas were averaged to 6.32 ± 4.35 spikes/s. The similar analysis procedure was applied to four-channel electrophysiological signals recorded in hippocampus, while the display time was prolonged to 100 s considering the low firing frequency in this region (Figure 5b). Five typical spike types were discriminated with Channel 6 detecting two distinct types. The firing rate declined sharply to 0.09 ± 0.06 spikes/s compared with the region of cortex.

### 3.3. Concurrent Analysis of Glutamate Distribution and Spike Firing Rate Changes from Cortex to Hippocampus

**Figure 6:**
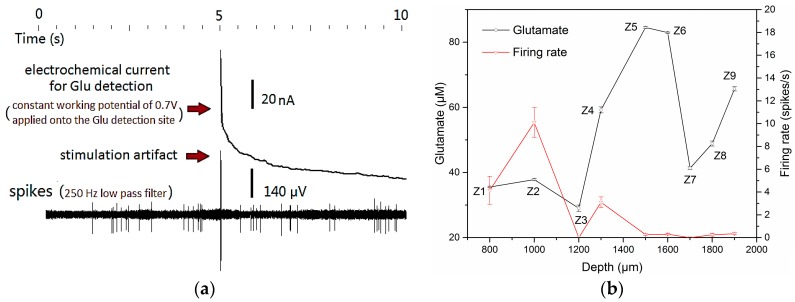
(**a**) Cross-talk evaluation between the dual mode signals of electrochemical currents and neural spikes in mouse cortex; (**b**) Glu concentration distribution (black line, **Left** *y*-axis) from cortex to hippocampus at nine numbered depths in the brain, and average firing rate (red line, **Right** *y*-axis) of simultaneously detected spikes.

To achieve efficient and reliable concurrent dual mode recording, avoiding cross-talk was the primary issue to be considered. Through careful design of the microelectrode spacing, reasonable setting of the electrochemical three-electrode-system, good shielding and grounding, and signal identification processing, the cross-talk between neural electrical and neural chemical signals was reduced to a minimum. As shown in Figure 6a, in a cross-talk evaluation test in vivo, one microelectrode on the MEA was first located close to an active neuron in mouse cortex to record stable spikes firings, then the constant potential of 0.7 V (DC voltage) was applied onto the adjacent electrochemical working electrode to start Glu detection. Except an obvious high-frequency stimulation artifact, no severe cross-talk between the dual mode signals was observed. The spike-firing rate before and after applying the working potential was almost the same, and the electrochemical detection currents would not fluctuate with the burst firing of the spikes. That means there was no current flow from the electrophysiological system to the electrochemical electrodes. Cross-talk could be well controlled during the process of simultaneous dual mode recording.

Figure 6b reveals the concurrent changes in Glu concentration and spike firing frequency during implantation. The numbers (*Z*1–*Z*9) beside data points were in accordance with the numbered recording depths in Figure 3. In the recording session at each numbered depth, the concentration of Glu was calculated from calibration curve and was adjusted by subtracting baseline current of 15.19 pA from the averaged oxidation current. The Glu concentrations were 35.5 μM, 37.8 μM at depth *Z*1, *Z*2 corresponding to the brain region of cortex, and ascended to 84.5 μM, 82.9 μM at depth *Z*5, *Z*6 located in hippocampus CA1 region. The two valley values at depth *Z*3, *Z*7 appeared at the transition regions of cortex and CA1 region, CA1 and the dentate gyrus (DG) region respectively. The dual-mode results revealed that Glu concentration in the layer of high-dense pyramidal neurons in hippocampus is roughly 50 μM higher than in Layer V of pyramidal neurons in cortex, while the firing rate of concurrently recorded spikes decline from 6.32 ± 4.35 spikes/s in cortex to 0.089 ± 0.058 spikes/s in hippocampus. It was found that high concentration of excitatory neurotransmitter Glu didn’t necessarily mean a high firing rate of concurrently recorded spikes.

In further study, similar dual mode recording processes will be conducted and statistically analyzed for different mice, including mice model with neurological diseases, to verify the above phenomenon and uncover the exact mechanisms. Despite the limited amount of mouse samples, the present results demonstrated the feasibility of the MEA probe for in situ, real-time, dual-mode monitoring of Glu and electrophysiological signals in mouse brain. Compared with the microdialysis probes, which were usually thicker than 500 μm and longer than 1 mm of the sensitive sampling area [20], the small size of our MEA caused less damage and achieved much higher spatial resolution of 10 μm (diameter of each recording site), which enabled the distinguishing of layered fine structures within hippocampus. Also, the dynamic changing process of the dual-mode signals across different brain tissues could be monitored in real time during the continuous implantation.

## 4. Conclusions

A novel implantable silicon-based MEA probe was designed and fabricated and the 16 microelectrodes were directionally modified with nano-Pt and GluOx-mPD polymer to form dual-mode recording sites. The MEA was implanted from the cortex to the hippocampus in mouse brain for in situ real-time monitoring of Glu and electrophysiological signals. It was found that the Glu concentration was roughly 50 μM higher than that in cortex, and the firing rate of concurrently recorded spikes declined from 6.32 ± 4.35 spikes/s in cortex to 0.089 ± 0.058 spikes/s in hippocampus. The present results demonstrated that the dual-mode MEA probe was capable in neurological detections in vivo with high spatial resolution and dynamical response, which laid the foundation of further pathology studies in hippocampus of mouse models with nervous or mental disorders.

## References

[1] Hascup K.N., Hascup E.R., Pomerleau F., Huettl P., Gerhardt G.A. (2008). Second-by-second measures of L-glutamate in the prefrontal cortex and striatum of freely moving mice. J. Pharmacol. Exp. Ther..

[2] Song Y., Lin N., Liu C., Jiang H., Xing G., Cai X. (2012). A novel dual mode microelectrode array for neuroelectrical and neurochemical recording in vitro. Biosens. Bioelectron..

[3] Tamminga C.A., Stan A.D., Wagner A.D. (2010). The hippocampal formation in schizophrenia. Am. J. Psychiatry.

[4] Andersen P., Bliss T.V.P., Skrede K.K. (1971). Unit analysis of hippocampal population spikes. Exp. Brain Res..

[5] Kanamori K. (2015). Disinhibition reduces extracellular glutamine and elevates extracellular glutamate in rat hippocampus in vivo. Epilepsy Res..

[6] El-Khoury R., Panayotis N., Matagne V., Ghata A., Villard L., Roux J.C. (2014). GABA and glutamate pathways are spatially and developmentally affected in the brain of Mecp2-deficient mice. PLoS ONE.

[7] Bianchi L., Ballini C., Colivicchi M. (2003). Investigation on acetylcholine, aspartate, glutamate and gaba extracellular levels from ventral hippocampus during repeated exploratory activity in the rat. Neurochem. Res..

[8] Mizuseki K., Royer S., Diba K. (2012). Activity dynamics and be havioral correlates of CA3 and CA1 hippocampal pyramidal neurons. Hippocampus.

[9] Blanche T., Spacek M., Hetke J. (2005). Polytrodes: High-density silicon electrode arrays for large-scale multiunit recording. J. Neurophysiol..

[10] Stephens M.L., Pomerleau F., Huettl P., Gerhardt G.A., Zhang Z. (2010). Real-time glutamate measurements in the putamen of awake rhesus monkeys using an enzyme-based human microelectrode array prototype. J. Neurosci. Methods.

[11] Johnson M.D., Franklin R.K., Gibson M.D., Brown R.B., Kipke D.R. (2008). Implantable microelectrode arrays for simultaneous electrophysiological and neurochemical recordings. J. Neurosci. Methods.

[12] Jason J., Karen M., Greg G. (2000). Ceramic-Based Multisite Microelectrodes for Electrochemical Recordings. Anal. Chem..

[13] Jason J., Greg G. (2001). Self-Referencing Ceramic-Based Multisite Microelectrodes for the Detection and Elimination of Interferences from the Measurement of L-Glutamate and Other Analytes. Anal. Chem..

[14] Wei W., Song Y., Wang L., Zhang S., Luo J., Xu S., Cai X. (2015). An implantable microelectrode array for simultaneous L-glutamate and electrophysiological recordings in vivo. Microsyst. Nanoeng..

[15] Wei W.J., Song Y.L., Shi W.T., Liu C.X., Jiang T.J., Cai X.X. (2013). A novel microelectrode array probe integrated with electrophysiology reference electrode for neural recording. Key Eng. Mater..

[16] Wei W., Song Y., Fan X., Zhang S., Wang L., Xu S., Wang M., Cai X. (2016). Simultaneous recording of brain extracellular glucose, spike and local field potential in real time using an implantable microelectrode array with nano-materials. Nanotechnology.

[17] Zhang S., Song Y., Wang M., Zhang Z., Fan X., Song X., Zhuang P., Yue F., Chan P., Cai X. (2016). A silicon based implantable microelectrode array for electrophysiological and dopamine recording from cortex to striatum in the non-human primate brain. Biosen. Bioelectron..

[18] Mizuseki K., Diba K., Pastalkova E. (2011). Hippocampal CA1 pyramidal cells form functionally distinct sublayers. Nat. Neurosci..

[19] Arnthjensen N., Jabaudon D., Scanziani M. (2002). Cooperation between independent hippocampal synapses is controlled by glutamate uptake. Nat. Neurosci..

[20] Fedele E., Mazzone P., Stefani A., Bassi A., Ansaldo M., Raiteri M. (2001). Microdialysis in parkinsonian patient basal ganglia: Acute apomorphine-induced clinical and electrophysiological effects not paralleled by changes in the release of neuroactive amino acids. Exp. Neurol..

